# Pennes’ bioheat equation vs. porous media approach in computer modeling of radiofrequency tumor ablation

**DOI:** 10.1038/s41598-021-84546-6

**Published:** 2021-03-05

**Authors:** Claudio Tucci, Macarena Trujillo, Enrique Berjano, Marcello Iasiello, Assunta Andreozzi, Giuseppe Peter Vanoli

**Affiliations:** 1 Dipartimento Di Medicina E Scienze Della Salute “Vincenzo Tiberio”, Università del Molise, Via Francesco De Sanctis 1, 86100 Campobasso, Italy; 2 BioMIT, Department of Applied Mathematics, Universitat Politècnica de València, 46022 Camino de Vera, Valencia Spain; 3 BioMIT, Department of Electronic Engineering, Universitat Politècnica de València, 46022 Camino de Vera, Valencia Spain; 4 Dipartimento Di Ingegneria Industriale, Università Degli Studi Di Napoli Federico II, P.le Tecchio 80, 80125 Napoli, Italy

**Keywords:** Biomedical engineering, Computational biology and bioinformatics, Biological techniques, Computational science

## Abstract

The objective of this study was to compare three different heat transfer models for radiofrequency ablation of in vivo liver tissue using a cooled electrode and three different voltage levels. The comparison was between the simplest but less realistic Pennes’ equation and two porous media-based models, i.e. the Local Thermal Non-Equilibrium (LTNE) equations and Local Thermal Equilibrium (LTE) equation, both modified to take into account two-phase water vaporization (tissue and blood). Different blood volume fractions in liver were considered and the blood velocity was modeled to simulate a vascular network. Governing equations with the appropriate boundary conditions were solved with Comsol Multiphysics finite-element code. The results in terms of coagulation transverse diameters and temperature distributions at the end of the application showed significant differences, especially between Pennes and the modified LTNE and LTE models. The new modified porous media-based models covered the ranges found in the few in vivo experimental studies in the literature and they were closer to the published results with similar in vivo protocol. The outcomes highlight the importance of considering the three models in the future in order to improve thermal ablation protocols and devices and adapt the model to different organs and patient profiles.

## Introduction

Interest in thermal ablation as an anticancer technique has grown through the years since the clinical data supports its use against different types of tumor, especially liver tumors, and it is a minimally invasive technique with certain advantages, such as shorter hospital stays and lower costs^1,2^. Different types of energy can be used to raise tissue temperature over 50 °C, such as radiofrequency (RF), microwaves or ultrasound to completely destroy tumor tissue while saving the surrounding healthy tissue. In this context, the mathematical modeling of heat transfer in biological tissue during thermal ablation has a key role to play in predicting coagulation zone volume (i.e. thermal lesion size)according to different factors, such as tumor size and shape. The results thus obtained may also give rise to the development of new medical devices and protocols for thermal ablation.

The importance of computational modeling in this field is due to the few in vivo studies carried out, which are in general expensive and time-consuming.

Various bioheat models have been developed since 1948, when Pennes proposed the simplest bioheat equation^3^ which is still widely used for its simplicity but nevertheless presents certain shortcomings. As it assumes a uniform perfusion rate, it does not consider blood flow direction and also neglects the artery-vein countercurrent arrangement. It also assumes that venous blood is the only type in thermal equilibrium with the tissue and that arterial blood remains at a constant temperature of 37 °C. Some researchers have tried to overcome these limitations by proposing different models to provide more accurate results. An in-depth review of the different bioheat models applied to hyperthermia treatments can be found in Andreozzi et al.^4^.

In this study, we focused on modeling liver radiofrequency ablation (RFA) and compared the performance of Pennes’ bioheat equation with other bioheat equations based on porous media theory in terms of coagulation zones and temperature distributions. According to the porous media theory, the entire biological medium can be divided into two distinct phases: the tissue phase, which is the solid part made up of cells and interstitial spaces, and the blood phase, i.e. the fluid part, which is represented by the blood flowing through the solid phase^5^. As will be explained below, the governing equations for the bioheat transfer and blood flow are thus averaged on a control volume which represents a fluid-saturated porous medium infiltrated by flowing blood. The blood volume fraction in the whole biological medium is represented by porosity, so the higher the vascularization of the tissue the more important will be its role in the model, such as in the liver, kidney, and tumors themselves.

**Figure 1:**
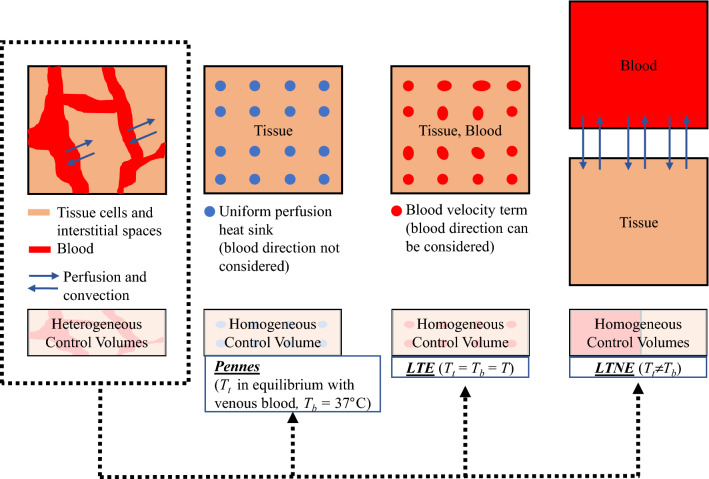
Differences between Pennes’ equation and porous media-based models.

Our motive for this study was that more realistic models based on porous media could predict tissue temperature more accurately, especially in highly porous organs such as the liver^6^. In addition, since the porous media theory includes fewer assumptions than other bioheat equations, it is reasonable to think that it is more suitable for treating heat transfer in biological tissues^5,7–11^. In fact in the models based on porous media, tissue and blood temperatures are calculated separately, ignoring Pennes’ assumption of uniform 37 ºC blood temperature throughout the tissue, which is unrealistic in thermal ablation, where tissue temperatures can go beyond 100 °C near the probe. The porous media theory also considers blood flow in different directions to represent the real vascular structure, so that the other strong assumption of uniform perfusion is not included. The differences between Pennes’ equation and the porous media-based models described above are graphically illustrated in Fig. 1.

We considered two different models based on porous media theory: the Local Thermal Equilibrium equation (LTE) and the Local Thermal Non-Equilibrium equations (LTNE)^5^. In both models we modified the governing equations to consider vaporization in both tissue and blood and assumed uniform blood velocity in all directions to simulate a more realistic blood flow in a vascular network. All the models built were designed for the particular case of in vivo liver RFA with a cooled electrode and an impedance-controlled pulsing protocol as described in^12^, which represents the usual clinical scenario in hepatic tumor RFA^13^.

## Results and discussion

### Analysis of outcomes

**Figure 2:**
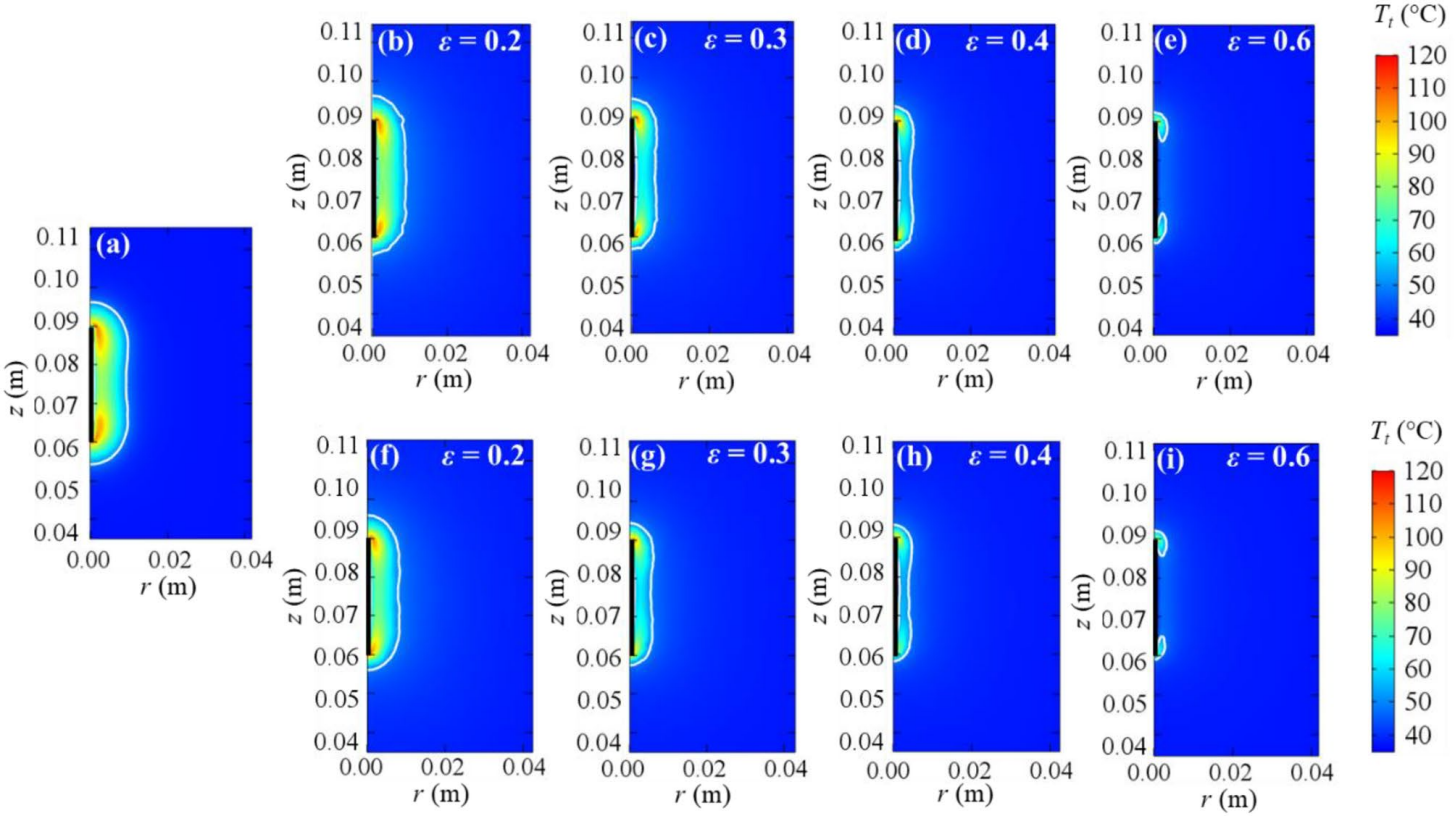
Tissue temperature distributions after 720 s of applying 45 V computed from Pennes’ bioheat model (**a**), LTE model (**b**–**e**), and LTNE model (**f**–**i**) for different porosity values (*ɛ*). White line represents coagulation zone contour. The thicker black line identifies the position of the electrode.

**Figure 3:**
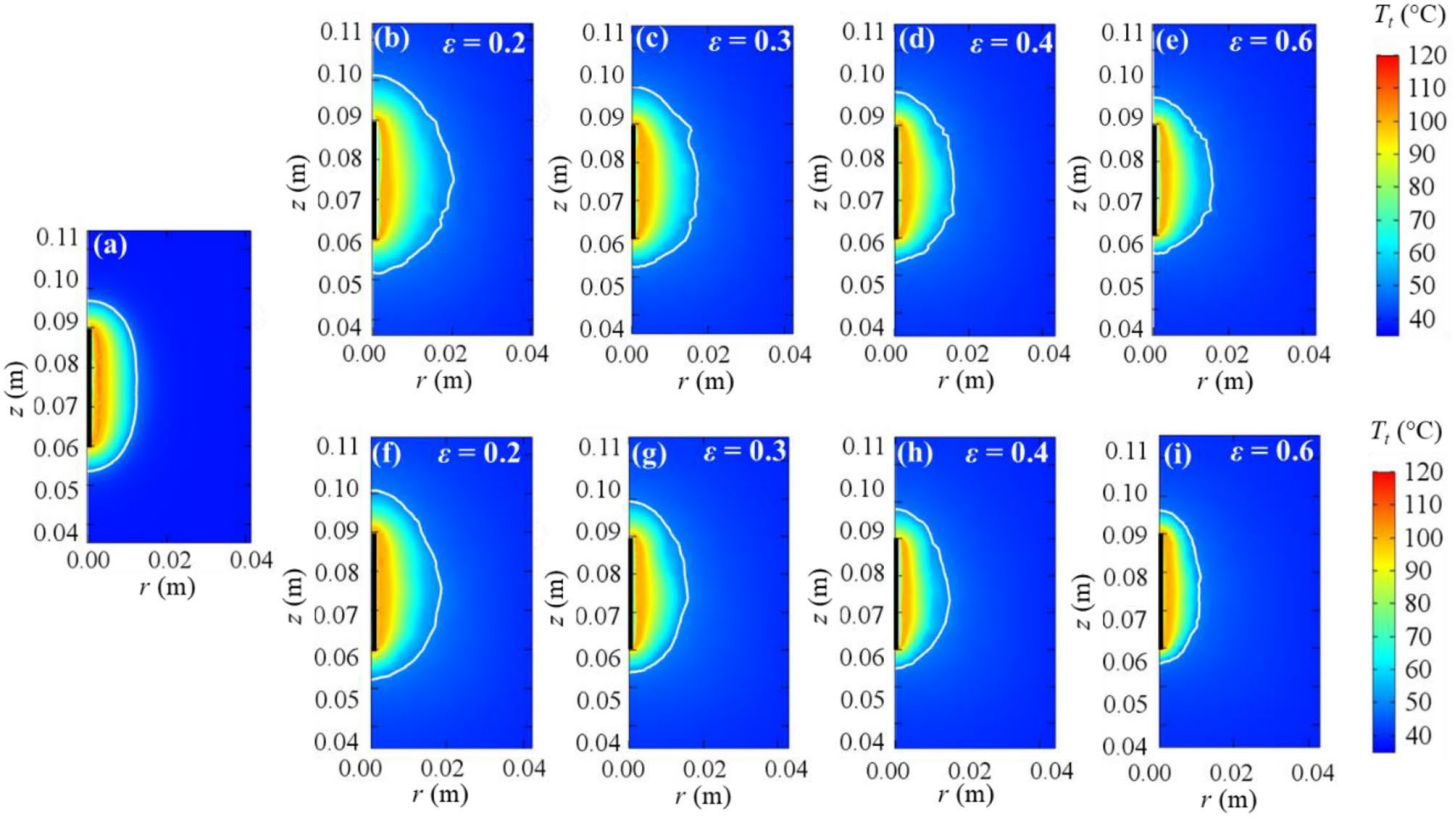
Tissue temperature distributions after 720 s of applying 65 V computed from Pennes’ bioheat model (**a**), LTE model (**b**–**e**), and LTNE model (**f**–**i**) for different porosities (*ɛ*). White line represents coagulation zone contour. The thicker black line identifies the position of the electrode.

The results are given in terms of the minor diameter of coagulation zones *d*_*c*_ (transverse diameter to the applicator shaft in *r* direction in Figs. 2 and 3), total energy delivered during the application *E*_*RF*_, maximum tissue temperature reached *T*_*t,*max_, total time in which the generator is “on” (*t*_on_), time of first roll-off (*t*_*roll-off*_) and number of roll-offs *N*_*roll-off*_, distinguishing between the Pennes’, LTNE and LTE results with four different porosity values.

All the models employed were compared at voltage values of 45, 65 and 90 V for720 s. As expected, only in the 45 V case did the absence of roll-offs allow RF power to be applied continuously for the entire 720 s. When roll-offs occurred at 65 and 90 V the pulsing protocol was activated^12^.

### Low voltage RFA

Table 1 shows the results of the 45 V simulations. The first finding was that the coagulation diameters computed from the LTE and LTNE models were smaller than those from the Pennes’ model for all porosity values. For instance, the difference between Pennes’ and LTE ranged from only 1.30 × 10^–3^ m for *ε* = 0.2 to 12.4 × 10^–3^ m for *ε* = 0.6. The coagulation diameter reduced dramatically as porosity increased (from 0.2 to 0.6) and the value of the maximum temperature in tissue also dropped. In fact, the higher the porosity, the larger the convective contribution of the mass blood flux.

**Table 1:** 45 V RFA results computed for different bioheat models (Pennes’, Local Thermal Equilibrium, Local Thermal Non-Equilibrium) and porosity values.

Model	*ε*	*d*_*c*_ (m )	*E*_*RF*_ (kJ)	*t*_*on*_ (s)	*t*_*roll-off*_ (s)	*N*_*roll-off*_	*T*_*t,*max_ (°C)
Pennes	**-**	1.94 × 10^−2^	19.7	720	–	–	106
LTE	0.2	1.81 × 10^−2^	19.5	720	–	–	106
	0.3	1.30 × 10^−2^	18.6	720	–	–	105
	0.4	1.01 × 10^−2^	17.7	720	–	–	101
	0.6	7.00 × 10^−3^	16.7	720	–	–	87
LTNE	0.2	1.69 × 10^−2^	19.4	720	–	–	105
	0.3	1.23 × 10^−2^	18.2	720	–	–	103
	0.4	9.40 × 10^−3^	17.4	720	–	–	94
	0.6	6.50 × 10^−3^	16.5	720	–	–	81

*d*_*c*_: coagulation diameter; *E*_*RF*_: applied RF energy; *t*_*on*_: total time generator is “on”; *t*_*roll-off*_: time of first roll-off; *N*_*roll-off*_: number of roll-offs.

At lower temperatures electrical conductivity increased less, so impedance (*Z*) decreased less and RF applied power decreased too (*P* = *V* ^2^/*Z*). This was confirmed by the reduced energy (~ 19.5 kJ to 16.5 kJ when porosity rose from 0.2 to 0.6). This highlights the importance of considering the blood volume fraction, (i.e. porosity) in bioheat thermal ablation models. The porosity of different organs ranges from negligible values such as the brain (0.03 to 0.05) to very high as in liver (about 0.3) and kidney (about 0.35)^6,14–19^.

The same organ can have different porosities in different physiological conditions, as happens for example in chronic liver hepatitis and cirrhosis, when porosity can be less than 0.2^20^. Figure 2 shows temperature distributions and coagulation contours at 45 V and 720 s computed from the Pennes’, LTE and LTNE models, respectively. The mean temperature dropped in the domain as porosity increased as given in Fig. 2.

The results of the LTE and LTNE models at the same porosity value were almost identical. As all the cases referred to a tissue with infiltrating terminal arteries, as described in Section "Modified LTNE model", the values of blood vessel diameter and blood velocity in the LTNE model were small enough to validate the local thermal equilibrium assumption, in agreement with the results reported in^7,21,22^, which all confirmed that the LTE temperature distributions agree with those of LTNE only when small blood vessels are considered (up to 3.00 × 10^–5^ m) and blood velocity is less than 4.00 × 10^–3^ m s^-1^, showing that blood does not act as a heat sink in these cases. Note that the LTNE and LTE equations are not limited to modeling vessels smaller than 1 mm, but can also model larger vessels, which were not taken into account in this work.

### Medium and high voltage RFA (65 and 90 V)

Tables 2 and 3 show the results for 65 and 90 V, respectively. Unlike 45 V, the coagulation diameters computed from the LTE and LTNE models with 65 and 90 V at all porosity values were greater than those from the Pennes’ model, except for LTNE *ε* = 0.6. This can be explained by their different ways of applying power: at 45 V it was continuous for 720 s, so that the only physical phenomenon that affected coagulation zone size was the larger heat loss through blood as porosity increased, which also meant less energy delivered in the LTNE and LTE models. Instead, the higher voltage values involving alternating periods of rising (power on) and falling (power off) temperatures weighed on the different thermal inertia of the models. In fact, unlike Pennes’ equation, they considered solid and fluid phases separately at different temperatures and water contents, which resulted in a better heat storage capability in the “off” periods. Consequently, the higher heat storage capability became determinant in achieving necrosis, especially away from the electrode, so larger coagulation diameters were finally obtained. This can also be seen in Fig. 5 concerning the comparison with experimental results.

**Table 2:** Results for different bioheat models at 65 V.

Model	*ε*	*d*_*c*_ (m)	*E*_*RF*_ (kJ)	*t*_*on*_ (s)		*t*_*roll-off*_ (s)		*N*_*roll-off*_	*T*_*t,*max_ (°C)
Pennes	**–**	2.47 × 10^–2^	30.7	494		126		16	114
LTE	0.2	4.09 × 10^–2^	28.6	437		127		19	113
	0.3	3.46 × 10^–2^	30.2	464		135		18	112
	0.4	3.21 × 10^–2^	30.3	475		151		17	112
	0.6	3.17 × 10^–2^	32.1	514		178		14	112
LTNE	0.2	3.72 × 10^–2^	31.2	480		131		16	113
	0.3	3.17 × 10^–2^	31.8	495		145		15	112
	0.4	3.02 × 10^–2^	32.2	511		164		14	113
	0.6	2.23 × 10^–2^	34.2	555		209		11	113

*d*_*c*_: coagulation diameter; *E*_*RF*_: applied RF energy; *t*_*on*_: total time generator is “on”; *t*_*roll-off*_: time at first roll-off; *N*_*roll-off*_: number of roll-offs.

**Table 3:** Results for different bioheat models at 90 V.

Model	*ε*	*d*_*c*_ (m)	*E*_*RF*_ (kJ)	*t*_*on*_ (s)	*t*_*roll-off*_ (s)	*N*_*roll-off*_	*T*_*t,*max_ (°C)
Pennes	**-**	2.50 × 10^–2^	36.5	270	31	30	119
LTE	0.2	4.13 × 10^–2^	33.8	239	31	32	118
	0.3	3.42 × 10^–2^	34.9	251	35	32	119
	0.4	3.28 × 10^–2^	35.3	254	36	31	118
	0.6	3.16 × 10^–2^	36.2	270	38	30	119
LTNE	0.2	3.94 × 10^–2^	38.1	270	34	30	118
	0.3	3.47 × 10^–2^	38.8	281	35	30	120
	0.4	2.99 × 10^–2^	38.6	286	36	29	117
	0.6	2.62 × 10^–2^	39.8	300	40	28	118

*d*_*c*_: coagulation diameter; *E*_*RF*_: applied RF energy; *t*_*on*_: total time generator is “on”; *t*_*roll-off*_: time at first roll-off; *N*_*roll-off*_: number of roll-offs.

As for 45 V, at these voltages the larger the porosity (from 0.2 to 0.6), the smaller the coagulation diameter (from 4.09 × 10^–2^ m to 3.17 × 10^–2^ m for LTE, and 3.72 × 10^–2^ m to 2.23 × 10^–2^ m for LTNE at 65 V, from 4.13 × 10^–2^ m to 3.16 × 10^–2^ m for LTE, and 3.94 × 10^–2^ m to 2.62 × 10^–2^ m for LTNE at 90 V).

Interestingly, the smaller diameter was associated with a slight increase in delivered energy: from 28.6 to 32.1 kJ for LTE and from 31.2 to 34.2 kJ for LTNE at 65 V, and 33.8 to 36.2 kJ for LTE and 38.1 to 39.8 kJ for LTNE at 90 V. This was probably due to roll-offs again, involving different thermal inertia in the LTE and LTNE models.

When porosity is increased, the larger blood volume removes heat from the tissue more effectively, which simultaneously delays roll-off, i.e. power can be applied longer (higher values of *t*_*on*_), requiring slightly higher power in LTE and LTNE.

The maximum tissue temperature was quite similar in all cases at the same voltage level (~ 114 ºC for 65 V and ~ 120 ºC for 90 V) due to the fact that in all cases the on–off periods avoided the temperature rising above the limit value.

When the LTE and LTNE models were compared at the same porosity value, the LTNE coagulation diameters were similar to or even smaller, with longer *t*_*on*_ and higher energy, possibly because of the effect of vaporization at different temperatures for tissue and blood, which is more significant for these applications than at 45 V.

For the same bioheat model, the results obtained at the two different voltage levels were almost identical, (differences in coagulation diameter from 1.00 × 10^–4^ m to 7.00 × 10^–4^ m in LTE and from 3.00 × 10^–4^ m to 3.90 × 10^–3^ m for LTNE), which suggests that 65 V is enough to obtain maximum coagulation diameter after 12 min. Figure 3 shows temperature distributions and coagulation contours at 65 V and 720 s computed from the Pennes’, LTE and LTNE models, respectively. Comparing Figs. 2 and 3 shows the different coagulation shapes at voltages higher than 45 V. In fact, at 65 V the zones are more spherical for both the LTE and LTNE models than Pennes’, especially at low porosities.

**Figure 4:**
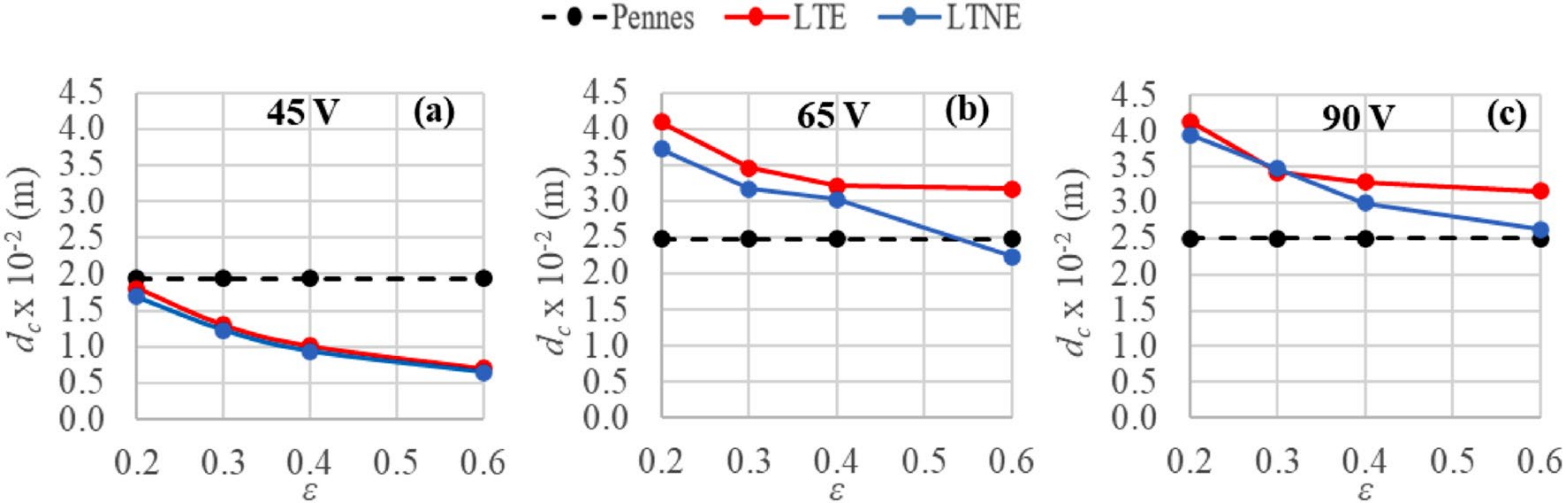
Transverse diameters of coagulation zone (*d*_*c*_) computed after 720 s of RFA for the three considered bioheat models (Pennes’, LTE and LTNE) at different porosity values (ε) and applied voltages: 45 V (**a**), 65 V (**b**) and 90 V (**c**).

Figure 4 summarizes the coagulation zone diameters computed from the three bioheat models at different porosities (models based on porous media approach).

As highlighted in Figs. 2, 3, and 4, the differences in terms of coagulation diameters and temperature distributions could differ significantly or not at all between the porous media-based and Pennes’ models, according to applied voltage and porosity. For instance, at 45 V, Pennes’ provides the same result as LTE and LTNE for *ε* = 0.2. In fact, the range of differences in coagulation diameters obtained is from only 1 mm at 45 V and LTE *ε* = 0.2 to about 1.60 × 10^–2^ m for the highest voltage applied and LTE *ε* = 0.2. These differences could play a relevant role in predicting coagulation zones, since the risk could be either incomplete ablation and tumor recurrence or overestimating the ablated area and healthy tissue necrosis.

### Comparison with experimental results

As regards the comparison of our computer results with*in vivo* experimental studies in the literature, Goldberg et al.^23^ reported a coagulation diameter of 3.70 × 10^–2^ ± 6.00 × 10^–3^ m for a pulsed protocol similar to the 90 V case. This value is in agreement with the results from the LTE and LTNE models with *ɛ* = 0.3 (which is the most realistic published values ^20,24–26^), i.e. 3.42 × 10^–2^ m and 3.47 × 10^–2^ m respectively, as shown in Table 3. Note that the exact protocol is unknown for the specific case, but the predominant current peak ofabout 1600 ± 71 mA is comparable with the 1500 mA predominant peak obtained in our case. Although these differences could slightly affect the results, they did not affect the overall comparison of all the models.

**Figure 5:**
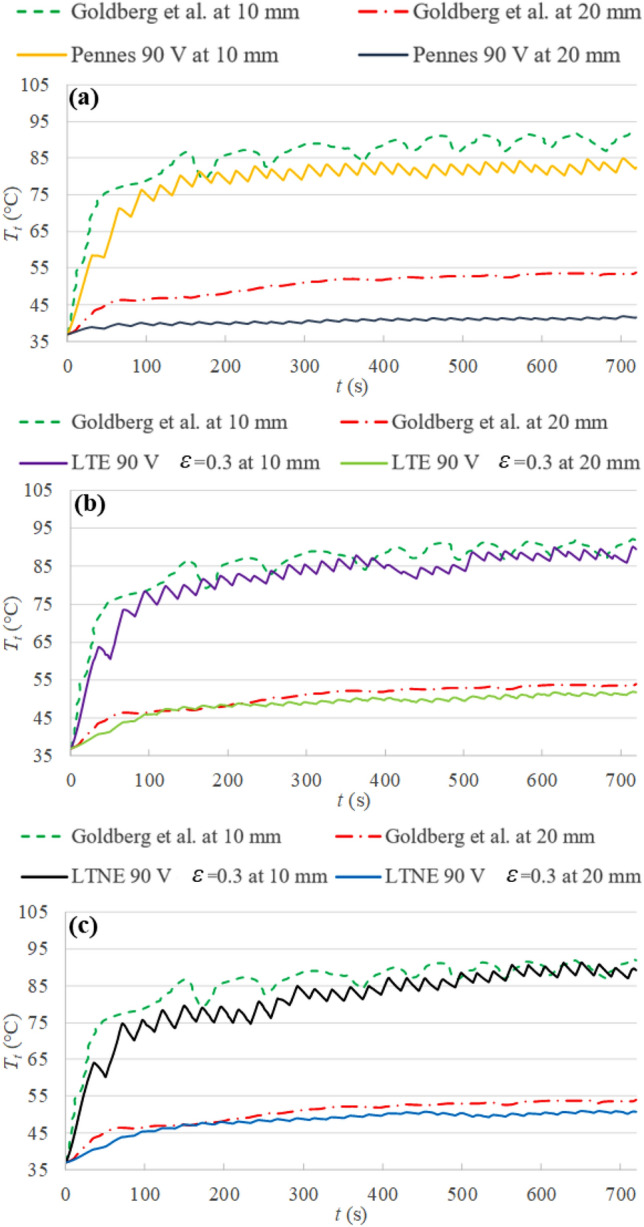
Temperature profiles for (**a**) Pennes’ equation, (**b**) LTE equation with *ɛ* = 0.3, (c**)** LTNE equations with *ɛ* = 0.3, and in vivo experimental results obtained by Goldberg et al.^23^.

Figure 5 compared the tissue temperature evolution at 10 mm and 20 mm from the electrode between the experimental results in^23^ and the three bioheat models: Pennes’ equation, LTE model with *ɛ* = 0.3, and LTNE model with *ɛ* = 0.3, respectively.

It can be seen that the temperature difference between the porous media-based models and the experimental data are ~ 4 °C and 2 °C at 10 mm and 20 mm, respectively. However the slightly higher predominant current peak could partially justify this difference. At 10 mm the temperature differences between the two porous media-based models and Pennes’ equation are only about 2 °C, mostly in the last 200 s, so the three bioheat models come reasonably close to the experimental data. However, at 20 mm, the differences between both the LTE and LTNE models and Pennes’ equation are ~ 8 °C, which justifies the difference of about 0.01 m in the final coagulation diameters obtained and suggests that the porous media-based models could be more accurate than Pennes’ in this case. The LTE and LTNE model results confirmed that the thermal local equilibrium is maintained in this case, as explained previously, so that they also give very similar outcomes in terms of tissue temperature.

In other studies with different setups, Solazzo et al.^27^ obtained a coagulation diameter of 3.60 × 10^–2^ ± 2 × 10^–3^ m with a high power generator, Song et al.^28^ achieved two transverse diameters of 1.85 × 10^–2^ ± 2.6 × 10^–3^ m and 2.18 × 10^–2^ ± 2.40 × 10^–3^ m, quite similar to our Pennes’ results, but we have no information about the voltage or current level and protocol used. Finally, Lee et al*.*^29^ reported coagulation diameters from 2.60 × 10^–2^ ± 4.00 × 10^–3^ m to 3.00 × 10^–2^ ± 8.00 × 10^–3^ m, which is a range covering our Pennes’ and porous media-based model results.

### Limitations of the study

The main limitation of this study is the lack of accurate experimental data on in vivo studies with which to compare our results, which means it is impossible to assess which model offers the best prediction of coagulation zone size in all conditions. By taking the currently available data into account, we found that LTNE and LTE (which must be considered more realistic bioheat models than Pennes) provided results in general within the range of values reported for experimental studies and approached the experimental data with similar protocols. As new values are reported in the future, it will be possible to determine which model best predicts thermal behavior during RFA and under what conditions.

Uniform porosity was assumed in the LTNE and LTE models, even though some studies showed porosity changes from the core to the rim of the tumor and in the adjacent normal tissue^26^. However, the same changing porosity could be implemented in both LTE and LTNE equations, so the comparison between the models would give the same conclusions.

The electrical conductivity used for tissue was for healthy liver tissue. Although it is known that inside a tumor its value can be slightly higher^30^ (0.45 S/m instead of 0.19 S/m at 37 °C), we consider that the effect of changing σ on the results would affect all three models equally, so that the conclusions would remain unchanged.

Finally, as the comparison was performed only for a single RF probe it does not seem likely that considering other RF applicator designs would change the conclusions in qualitative terms.

Overall, all these shortcomings should be solved in the future by means of developing experimental setups under strictly controlled conditions and with very accurate temperature measurements. From a computational point of view, the improvements could be focused on developing new models with variable porosity inside the tumor domain, i.e. from the center to the rim, and also considering differences in electrical conductivity between tumor and healthy tissues. All this would contribute to the development of realistic models tailored to specific organs and diseases.

## Conclusions

The aim of this work was to compare three different heat transfer models for radiofrequency ablation (RFA) of in vivo liver tissue using a cooled electrode. It is important to point out that our goal was not to suggest the best model for predicting the coagulation zone in RF tumor ablation, but to point out the differences obtained by comparing them. The study was conducted implementing a low voltage and two high voltage levels in order to consider cases with and without roll-off. As far as we know, this has never been done for in vivo liver thermal ablation. When the computer results obtained were compared with the few in vivo experimental works available in the literature it was found that they generally covered all the value ranges. However, for a similar protocol (e.g. 90 V pulsed protocol with a predominant current peak of about 1500 mA and 15 s off periods), the porous media model achieved a better agreement with the experimental results, since Pennes’ bioheat model tended to underestimate temperature distributions for the cases here investigated. Furthermore, from the theoretical point of view the porous media models represented a valid alternative approach that tried to overcome the constraints of Pennes' bioheat equation and could be useful when additional tissue characteristics (such as porosity) become available. Despite the paucity of in vivo studies and the need for further experimental studies, the three computer models should be taken into account in the future in order to go on improving both medical protocols and devices.

## Methods

### Model geometry and materials properties

**Figure 6:**
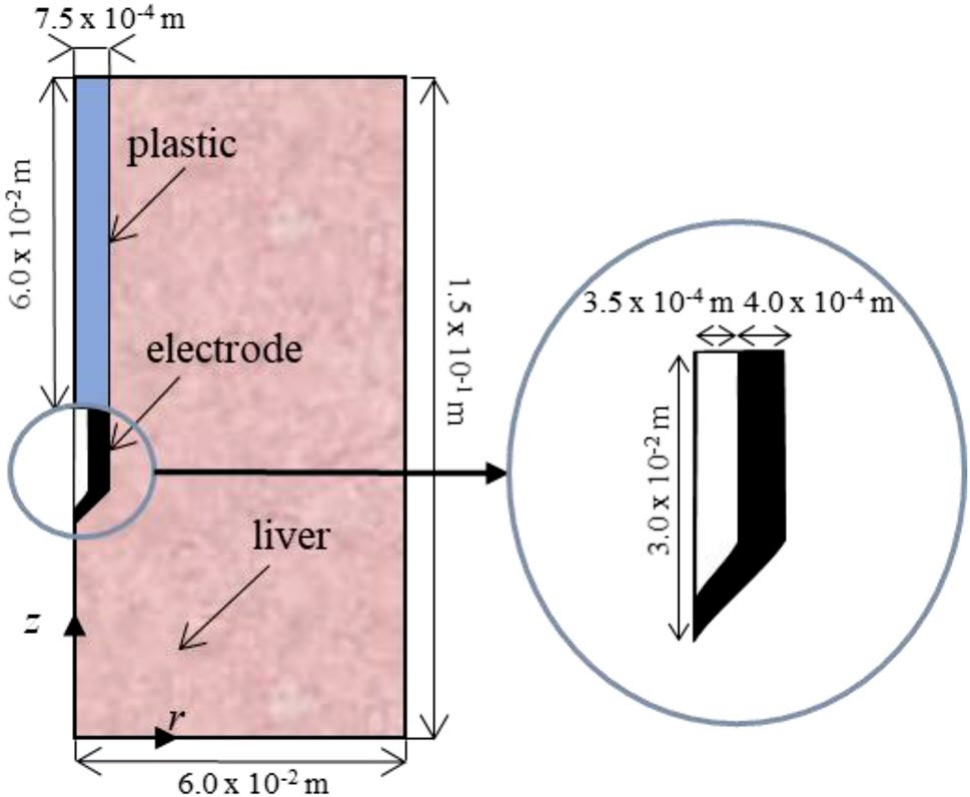
Two-dimensional axisymmetric model consisting of a fragment of liver tissue and an internally cooled electrode (out of scale).

Figure 6 shows the model geometry, which includes an RF applicator (comprised of metal electrode and plastic handle) completely inserted in the hepatic tissue. The needle-like electrode has a conical tip, 3.00 × 10^–2^ m long and 1.50 × 10^–3^ m outer diameter, and an internal cooling circuit with cold saline. The axial symmetry meant that a two-dimensional model could be implemented.

RFA is a monopolar procedure in which electrical current flows between the ablation electrode (see Fig. 6) and a large size dispersive electrode (in contact with the patient’s skin). The RF generator frequency is 500 kHz, and the electric and thermal properties of the materials used in the models are shown in Table 4. Since the temperature dependence of thermal conductivity is very small this was considered constant.

**Table 4:** Properties of the materials modeled^31–33^.

	*ρ* (kg m^-3^)		*c* (J kg^-1^ K^-1^)	*k* (W m^-2^ K^-1^)	*σ* (S m^-1^)
Liver	1080^a^		3455^a^	0.502	See Eq. (1)
	370^b^		2156^b^		
Blood	1000^a^		3639^a^	0.502	
	370^b^		2156^b^		
Electrode	8000		480	15	7.4 × 10^6^
Plastic	70		1045	0.026	10^–5^

^a^At temperatures below 100 °C, and ^b^ above 100 °C.

Electrical conductivity in tissue and blood was defined as a temperature-dependent piecewise function to consider the drastically reduced water content loss as temperature increases and vaporization occurs^34^.

1 $$\sigma (T) = \left\{ \begin{gathered} 0.19 \cdot e^{0.015(T - 37)} \quad 0 \, ^\circ {\text{C}} < T < 99 \, ^\circ {\text{C}} \hfill \\ 0.19 \cdot 2.5345 \quad 99 \, ^\circ {\text{C}} \le T \le 100 \, ^\circ {\text{C}} \hfill \\ 0.19 \cdot (2.5345 - 0.50183(T - 100)) \quad 100 \, ^\circ {\text{C}} < T \le 105 \, ^\circ {\text{C}} \hfill \\ 0.19 \cdot 2.5345 \cdot 10^{ - 2} \, T > 105 \, ^\circ {\text{C}} \hfill \\ \end{gathered} \right. \,$$

### Electrical problem equations

The heat source from RF power *Q*_*ext*_ (Joule losses) was given by:

2 $$Q_{ext} = \sigma\left| {\mathbf{E}} \right|^{{\mathbf{2}}}$$

where **E** is the electric field. **E** =  − ∇*V* was obtained from the governing equation of the electrical problem ∇·(σ(T) ∇*V* = 0), σ being the electrical conductivity and *V* the voltage.

A constant voltage was set on the ablation electrode during the entire protocol duration of 720 s except during roll-offs, when impedance rises by 30 Ω, in which case we followed the standard roll-off protocol of switching off the applied voltage for 15 s^12^.

The three voltage values employed were 45 V, 65 V and 90 V. This choice covers the range from the highest roll-off avoidance value (45 V) to the highest standard protocol value used in clinical practice (90 V)^12^.

For the electrical boundary conditions, an insulation electrical condition was applied to the contours of the tissue, except for top and bottom, which represent the dispersive electrode, where a condition *V* = 0 V was set.

### Thermal problem equations

**Figure 7:**
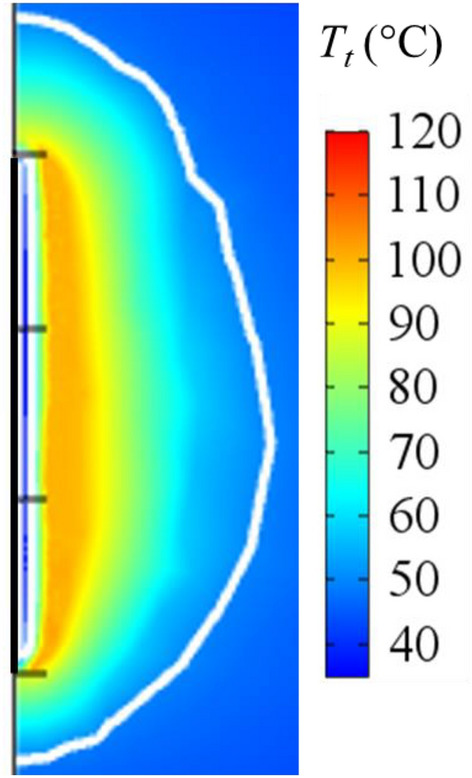
Tissue temperature distributions after 720 s of applying 90 V computed from LTNE model for *ɛ* = 0.3, a detail of the colder zone near the electrode. White line represents coagulation zone contour. The thicker black line identifies the position of the electrode.

Three different bioheat models were implemented: the Pennes’, LTNE and LTE. We first described the issues common to all three models. Initial and boundary tissue temperature were 37 °C. The thermal cooling effect inside the electrode is observable from the colder zone around the electrode in Fig. 7, where a detail of Fig. 3 is shown. This effect was modeled by applying a convective heat flux (*q*_*c*_) to the internal boundary of the electrode as follows:

3 $$q_{c} = h_{r} (T_{r} - T_{t} )$$

where *T*_*t*_ is the tissue temperature and *T*_*r*_ the coolant temperature (5 °C), *h*_*r*_ is the thermal convective coefficient (3127 W m^-2^ K^-1^), which was calculated by considering the electrode length (3.00 × 10^–2^ m) and a flow rate of 45 ml/min through an area equivalent to half the cross section of the inner diameter of the electrode, as described in^12^. The thermal damage was computed using the Arrhenius model, in which tissue damage is increased with time of exposure, and is obtained as follows from an exponential relationship between tissue exposure temperature, time and the kinetic parameters that account for morphologic changes in tissue relating to the thermal degradation of proteins^35^:

4 $$\Omega (t_{tot} ) = \int\limits_{0}^{{t_{tot} }} {Ae^{{ - \frac{\Delta E}{{RT_{t} (t)}}}} } dt$$

where *A* is the frequency factor (7.39 × 10^39^ s^−1^), *ΔE* is the activation energy for the irreversible damage reaction (2.577 × 10^5^ J mol^−1^)^36^, and *R* is the universal gas constant. The thermal damage contour was estimated using the isoline *Ω* = 4.6, which encompasses the zone with 99% cell death probability.

#### Pennes’ model

Pennes’ bioheat model was our starting point for the comparison of the models, based on the following equation:

5 $$\left( {\rho c} \right)_{t} \frac{{\partial T_{t} }}{\partial t} = \nabla \cdot (k_{t} \nabla T_{t} ) + Q_{perf} + Q_{met} + Q_{ext}$$

where subscript *t* refers to the tissue, *ρ* is the density, *c* the specific heat, *k*_*t*_ the thermal conductivity, *T*_*t*_ the temperature, *t* the time, *Q*_*perf*_ the blood perfusion term, *Q*_*met*_ the metabolic heat source (which is neglected in RFA applications^12^) and *Q*_*ext*_ is the external RF power density imposed to the tissue during RF power application (see Eq. (2)). To introduce vaporization into the Pennes’ equation, one alternative is to follow the enthalpy method as described in^31^, so that the term (*ρ*_*t*_*c*_*t*_) in Eq. (5) becomes:

6 $$(\rho c)_{t} = \left\{ \begin{gathered} (\rho _{l} c_{l} )_{t}\quad 0{\text{ }}^\circ {\text{C}} < T_{t} \le 99{\text{ }}^\circ {\text{C}} \hfill \\ \frac{{h_{{fg}} C_{{w,t}} }}{{\Delta T_{{b,t}} }}\quad 99{\text{ }}^\circ {\text{C}} < T_{t} \le 100{\text{ }}^\circ {\text{C}} \hfill \\ \rho _{g} c_{g} \quad T_{t} > 100{\text{ }}^\circ {\text{C}} \hfill \\ \end{gathered} \right.$$

where *ρ*_*l*_ and *c*_*l*_ are density and specific heat of tissue at temperature below 100 °C (liquid phase), *ρ*_*g*_ and *c*_*g*_ are density and specific heat of tissue at temperature above 100 °C (gas phase), *h*_*fg*_ is the product of water latent heat of vaporization and water density at 100 °C (2.17·10^9^ J m^-3^), and *C*_*w,t*_ is the water content inside the liver tissue (68%)^32^. Furthermore, Q_*perf*_ in Eq. (5) is defined as follows:

7 $$Q_{perf} = \beta \rho_{b} c_{b} \omega_{b} (T_{b} - T_{t} )$$

where *ρ*_*b*_ and *c*_*b*_ are the density and specific heat of blood respectively, *ω*_*b*_ is blood perfusion coefficient (0.019 s^-1^)^37^, *T*_*b*_ is blood temperature (which is assumed to be constant throughout the tissue and taken as body temperature of 37 °C in Pennes’ model), and *β* is a coefficient that is 0 or 1 depending on the value of thermal damage function *Ω* (see Eq. (4)), so, *β* = 0 for *Ω* ≥ 4.6 and *β* = 1 for *Ω* < 4.6.

#### Modified LTNE model

The second model was based on a modified form of the Local Thermal Non-Equilibrium equations, first developed in 1997 by Roetzel and Xuan^38^ to model heat transfer in a porous medium. Here, the entire biological medium is divided into two distinct phases: the tissue phase, which is the solid part, made up of cells and interstitial spaces, and the blood phase, which is the fluid part, represented by the blood flowing through the solid phase. We thus have two energy equations for this model, one for the tissue temperature (*T*_*t*_):

8 $$\left( {1 - \varepsilon } \right)\left( {\rho c} \right)_{t} \frac{{\partial \left\langle {T_{t} } \right\rangle }}{\partial t} = \left( {1 - \varepsilon } \right)k_{t} \nabla^{2} \left\langle {T_{t} } \right\rangle - h_{c} a\left( {\left\langle {T_{t} } \right\rangle - \left\langle {T_{b} } \right\rangle } \right) + \beta \rho_{b} c_{b} \omega (\left\langle {T_{b} } \right\rangle - \left\langle {T_{t} } \right\rangle ) + \left( {1 - \varepsilon } \right)Q_{ext}$$

and one for the blood temperature (*T*_*b*_):

9 $$\varepsilon \left( {\rho c} \right)_{b} \left( {\frac{{\partial \left\langle {T_{b} } \right\rangle }}{\partial t} + \beta \left\langle {\mathbf{u}} \right\rangle \cdot \nabla \left\langle {T_{b} } \right\rangle } \right) = \varepsilon k_{b} \nabla^{2} \left\langle {T_{b} } \right\rangle + h_{c} a\left( {\left\langle {T_{t} } \right\rangle - \left\langle {T_{b} } \right\rangle } \right) + \beta \rho_{b} c_{b} \omega (\left\langle {T_{t} } \right\rangle - \left\langle {T_{b} } \right\rangle ) + \varepsilon Q_{ext}$$

where *ɛ* is porosity (representing the blood volume/total volume ratio), **u** the blood velocity vector, *β* is the coefficient that is 0 or 1 depending on the value of thermal damage function *Ω* employed previously, *h*_*c*_ the interfacial heat transfer coefficient between tissue and blood phases, and *a* the volumetric heat transfer area between tissue and blood, which is computed from the definition of hydraulic diameter as *a* = 4·*ɛ*/*d*, where *d* is the blood vessel diameter. The second term on the right side of Eqs. (8) and (9) describes the interfacial convective heat transfer between blood and tissue phases across the vascular wall as defined by Newton’s cooling law. Note that the Pennes’ model does not consider any advective term such as the second term on the left side of Eq. (9), but an overall blood perfusion term as a heat sink for tissue. The LTE and LTNE models split the Pennes’ equation perfusion term into a modified perfusion term and a convective term^39^. The modified perfusion term in the porous media-based models (third term on the right side of Eqs. (8) and (9)) differs from Eq. (7) for the value of the perfusion coefficient *ω*, which is *ω* = 0.0005 s^-1^, instead of *ω*_*b*_ = 0.019 s^-1^, to consider the modification described above. In fact, Pennes obtained this coefficient for the volume flow of blood through tissue by fitting his model with experiments. He specified that 0.0005 s^-1^is the most suitable value when the balance between blood and tissue is incomplete. Nakayama et al*.* also used this value in their work on LTNE equations^3,39^.

The volume averaging technique is employed to consider the volume average quantities of the variables^40^, so that the symbol <  > refers to the average volume of a generic variable and is neglected from this point onwards. In the LTNE model, the most important modifications were to the phase change and blood velocity. In fact, the phase change was considered separately in both phases, while vaporization only refers to water in the tissue in the Pennes’ model (see Eq. 7). Vaporization was thus included as in Eq. (6) for the tissue phase, while for the blood phase we have:

10 $$(\rho c)_{b} = \left\{ \begin{gathered} {\text{ }}(\rho _{l} c_{l} )_{b} \quad 0{\text{ }}^\circ {\text{C}} < T_{b} \le 99{\text{ }}^\circ {\text{C}} \hfill \\ \frac{{h_{{fg}} C_{{w,b}} }}{{(100{\text{ }}^\circ C - 99{\text{ }}^\circ C)}}\quad 99{\text{ }}^\circ {\text{C}} < T_{b} \le 100{\text{ }}^\circ {\text{C}} \hfill \\ {\text{ }}\rho _{g} c_{g} \quad T_{b} > 100{\text{ }}^\circ {\text{C}} \hfill \\ \end{gathered} \right.$$

In this way the evaporation of water in blood was included, choosing the value of water content in blood *C*_*w,b*_ = 79% as in^41^. Four different blood velocity directions were considered to simulate a more realistic vascular network and the initial values were the same in all four directions *u*_*z*_ = *u*_*-z*_ = *u*_*r*_ = *u*_*-r*_, where directions *z* and *r* are specified in Fig. 6. As for blood perfusion, blood velocity was considered zero when cell death probability was 99% according to the *β* coefficient. Terminal artery blood velocity was chosen following the experimental values reported in Crezee and Lagendijk^42^, a reasonable choice according to Chen and Holmes’ LTNE model^43^, in which blood heat exchange is assumed to occur only downstream of the terminal arteries before the arterioles. Blood velocity value was thus assumed to be 4.00 × 10^–3^ m·s^-1^, with a blood vessel diameter *d* = 3.00 × 10^–5^ m. We also considered four different porosity values, *ɛ*: 0.2, 0.3, 0.4 and 0.6 to consider the liver values given in clinical and numerical studies in the literature^6,14,20,24,25^.These, in fact show wide dispersity because of the different assessment methods considered^20,25^.The interfacial heat transfer coefficient *h*_*c*_ was 170 W·m^-2^·K^-1^, as in Yuan^21^, based on experimental measurements. Table 5 summarizes the specific surface areas *a* and volumetric convective coefficients *h*_*v*_ for all the cases considered.

**Table 5:** Volumetric transfer areas and volumetric heat transfer coefficients considered in LTNE.

*a* (m^−1^)	*h*_*v*_ (W·m^−3^·K^−1^)
*ɛ* = 0.2 *ɛ* = 0.3 *ɛ* = 0.4 *ɛ* = 0.6	*ɛ* = 0.2 *ɛ* = 0.3 *ɛ* = 0.4 *ɛ* = 0.6
26,667 40,000 53,333 80,000	4.53E6 6.80E6 9.07E6 1.36E7

*a*: volumetric transfer area; *h*_*v*_: volumetric heat transfer coefficient.

#### Modified LTE equation

The LTNE model described above considered the blood phase and the tissue phase at two distinct temperatures (i.e. *T*_*t*_ ≠ *T*_*b*_). However, when the local thermal equilibrium hypothesis is maintained, tissue and blood are really at the same temperature, so that *T*_*t*_ = *T*_*b*_ = *T* and Eqs. (8) and (9) can be combined in a single equation as follows:

11 $$\left[ {\left( {1 - \varepsilon } \right)\left( {\rho c} \right)_{t} + \varepsilon \left( {\rho c} \right)_{b} } \right]\frac{\partial T}{{\partial t}} + \varepsilon \left( {\rho c} \right)_{b} \beta \cdot {\mathbf{u}} \cdot \nabla T = \left[ {\left( {1 - \varepsilon } \right)k_{t} + \varepsilon k_{b} } \right]\nabla^{2} T + Q_{ext}$$

In this model the heat sink effect for tissue is only represented by the advective term related to the blood velocity. In fact, when Eqs. (8) and (9) were combined the perfusion term disappeared. Note that in this case local thermal equilibrium should be a good approximation for temperature distributions because of the small size of the vessels considered (as described in^2,7,22^), while this assumption would not be valid in the presence of larger vessels. Even if the two phases are at the same temperature, they have different water contents, as in the LTNE model, so that vaporization was included as in Eqs. (6) and (10) by considering *T*_*t*_ = *T*_*b*_ = *T.* The assumptions on blood velocity for LTNE were also valid for this model.

### Numerical solution

The three models were numerically solved with the software Comsol Multiphysics (Burlington, MA, USA). A triangular mesh was employed with a finer size on the boundary between electrode and tissue domains as in^12^, where the highest temperature gradients take place. The grid convergence was verified on the maximum tissue temperature (*T*_*t,*max_) obtained at first roll-off time. When the difference between simulations was less than 0.5% in *T*_*t,*max_ we considered the former mesh size as appropriate. A similar convergence test was employed to estimate the optimal outer dimensions. The PARDISO direct solver was used with the implicit intermediate Backward Differentiation Formulas (BDF) time stepping method, where the intermediate configuration was chosen to fix the initial and maximum steps of the solver, in this case 0.01 s and 1 s, respectively.

## List of symbols

*A*: Volumetric transfer area (m^−^^1^)
*A*: Frequency factor (s^−^^1^)
*c*: Specific heat (J kg^-1^ K^−^^1^)
*C*_w_: Water content (%)
*d*: Diameter of blood vessel (m)
*d*_c_: Diameter of the coagulation zone (m)
**E**: Electric field vector (V m^-1^)
*E*_RF_: Applied radiofrequency energy (J)
*h*_*c*_: Convective interfacial heat transfer coefficient (W m^-2^ K^−^^1^)
*h*_fg_: Product of water latent heat of vaporization and water density at 100 °C (J m^−^^3^)
*h*_r_: Convective coefficient to model the electrode cooling system (W m^−^^2^ K^−^^1^)
*h*_v_: Volumetric convective heat transfer coefficient (W m^−^^3^ K^−^^1^)
*k*: Thermal conductivity (W m^−^^1^ K^−^^1^)
*N*_roll-off_: Number of roll-offs (–)
*P*: Applied power (W)
*q*_c_: Convective heat flux (W m^−^^2^)
*Q*_ext_: External power density (W m^−^^3^)
*Q*_met_: Metabolic power density (W m^−^^3^)
*Q*_perf_: Blood perfusion power density (W m^−^^3^)
*r*: Spatial coordinate (m)
*R*: Universal gas constant (J mol^-1^ K^−^^1^)
*t*: Time (s)
*t*_roll-off_: Time of first roll-off (s)
*T*: Temperature (K)
*T*_r_: Coolant temperature (K)
**u**: Velocity vector (m s^−^^1^)
*V*: Voltage (V)
*z*: Spatial coordinate (m)
*Z*: Impedance (Ω

## Greek symbols

*β*: Coefficient (–)
*ΔE*: Activation energy (J mol^−^^1^)
*ε*: Porosity (–)
*ρ*: Density (kg m^−^^3^)
*σ*: Electric conductivity (S m^−^^1^)
*ω*: Blood perfusion in LTNE model (s^−^^1^)
*ω*_*b*_: Blood perfusion in Pennes’ equation (s^−^^1^)
*Ω*: Thermal damage (–)

## Subscripts

*b*: Blood
*g*: Referred to temperatures above 100 °C
*l*: Referred to temperatures below 100 °C
max: Maximum
on: Voltage switched on
*t*: Tissue
tot: Total

## Abbreviations

BDF: Backward Differentiation Formulas
LTE: Local Thermal Equilibrium
LTNE: Local Thermal Non-Equilibrium
RF: Radiofrequency
RFA: Radiofrequency Ablation
